# A case of paraneoplastic pityriasis rubra pilaris

**DOI:** 10.1016/j.jdcr.2023.06.045

**Published:** 2023-07-13

**Authors:** Nina Mehta, Margaret M. Coates, J. Alex Miles, Jayson Miedema, Rachel C. Blasiak

**Affiliations:** a University of North Carolina School of Medicine, Chapel Hill, North Carolina; b Department of Dermatology, University of North Carolina School of Medicine, Chapel Hill, North Carolina

**Keywords:** lung carcinoma, oncology, paraneoplastic, pityriasis rubra pilaris, skin of color

## Introduction

Pityriasis rubra pilaris (PRP) is an uncommon, chronic papulosquamous disorder with an unestablished etiology.^1^ It is typically characterized by reddish orange scaly plaques, islands of sparring, palmoplantar keratoderma, and keratotic follicular papules.^1^ In darker skin types, the papules are violaceous to hyperpigmented.^2^ Infectious, autoimmune, and drug-related etiologies have been hypothesized.^3^ More recently, PRP has been reportedly associated with both solid and hematolymphoid malignancies and thus labelled paraneoplastic PRP (pPRP).^1^

PRP is often recalcitrant to treatment and there are no US Food and Drug Administration (FDA)-approved treatments. Typical therapies for mild PRP include emollients, keratolytic agents, topical corticosteroids, and topical calcineurin inhibitors. Systemic therapy including oral retinoids and methotrexate have also shown promise for managing symptoms and reducing inflammation in more severe cases. Biologics commonly used in the management of psoriasis, including tumor necrosis factor alpha (TNF alpha)-inhibitors, secukinumab, and ustekinumab have recently been used for treatment of PRP.^4^ Compared to traditional PRP, pPRP is less responsive to typical therapies and is particularly challenging to treat without addressing the underlying malignancy.^1^

Herein, we present a case of pPRP in a patient with adenosquamous carcinoma of the lung.

## Case presentation

**Fig 1:**
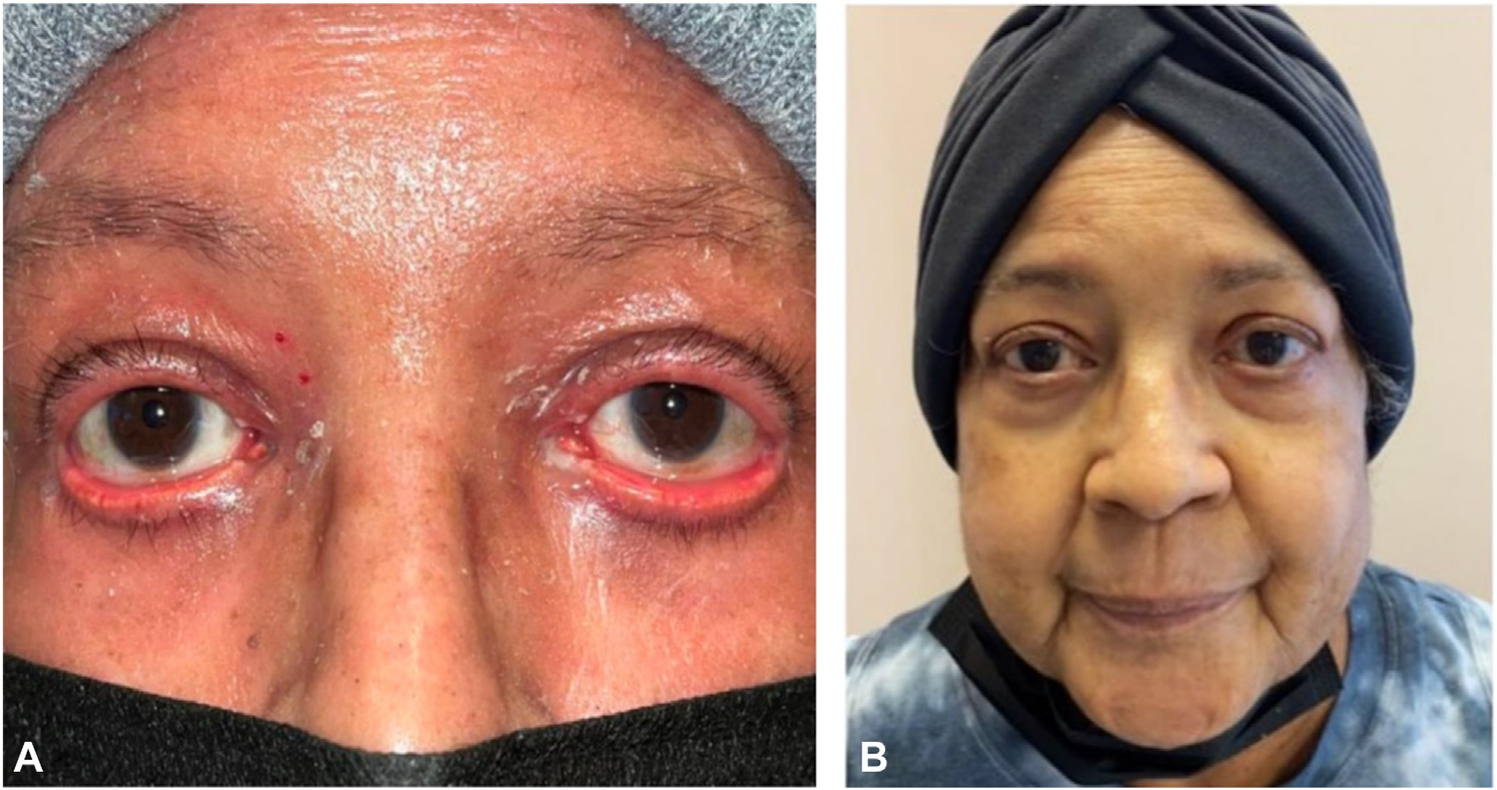
Facial Improvement. **A,** Appearance of face with ectropion before treatment. **B,** Resolution of ectropion and facial erythema after carcinoma resection and wet wraps.

**Fig 2:**
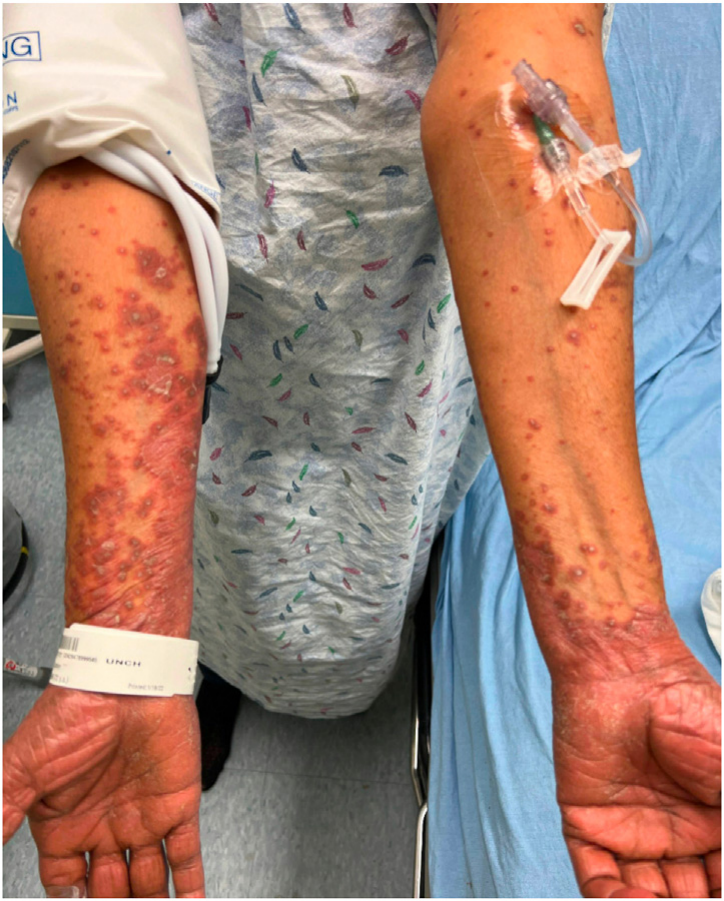
Palmar keratoderma. *Orange* to *pink* palmar hyperkeratosis with scale.

**Fig 3:**
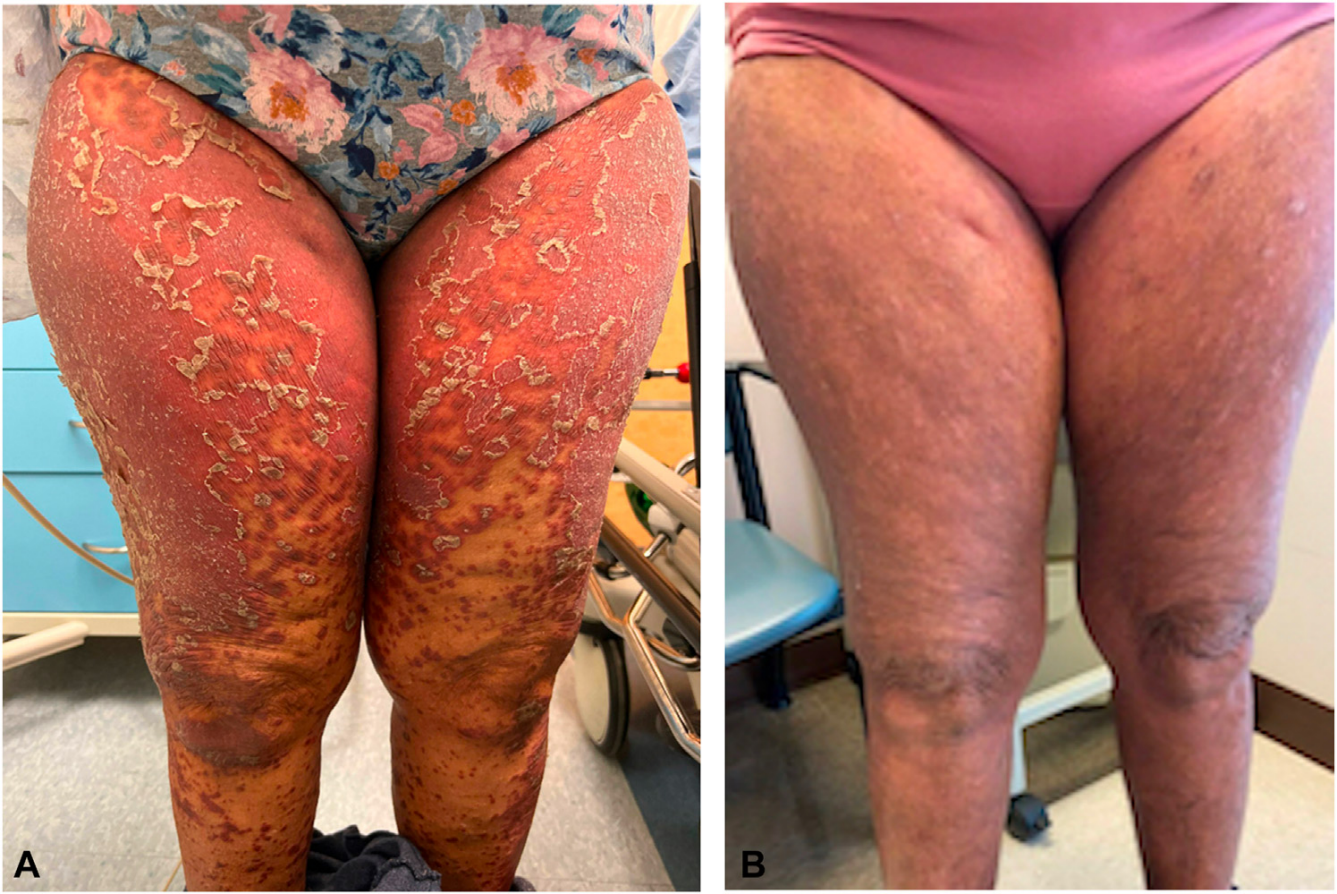
**A**, *Red* scaly plaques with islands of sparring. **B**, Resolution of scaly plaques with post-inflammatory hyperpigmentation.

A 72-year-old female with a history of hypertension and prior tobacco use initially presented to the dermatology clinic with a 1-month history of a new rash that began on her hands and spread proximally (Fig 1, Fig 2, Fig 3). The rash appeared following intramuscular steroid injections given for a chronic obstructive pulmonary disease exacerbation. Given the extensive body surface area involvement, pain associated with using her hands and feet, and development of ectropion, biopsy and laboratory screening were performed in preparation for potential use of systemic therapy. Differential diagnosis at initial presentation included psoriasis, PRP, dermatomyositis, and a psoriasiform drug eruption.

**Fig 4:**
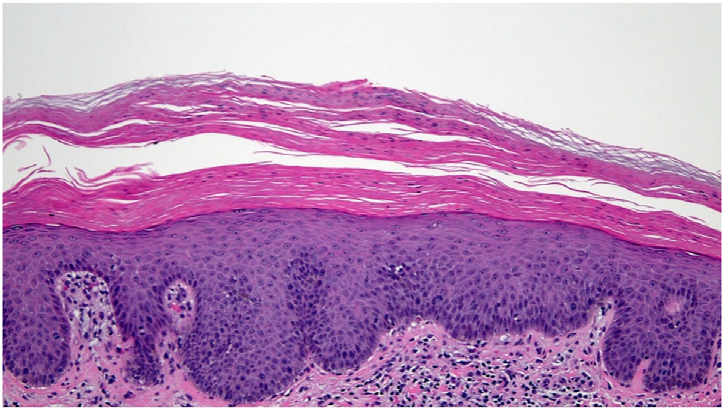
Histopathology. Psoriasiform epidermal hyperplasia with alternating ortho and parakeratosis and a lymphocytic infiltrate in the superficial dermis.

Her skin biopsy revealed psoriasiform epidermal hyperplasia with overlying hyperkeratosis and parakeratosis alternating with orthokeratosis both horizontally and vertically. The epidermis displayed spongiosis with a superficial vascular ectasia, while the superficial dermis revealed a sparse lymphocytic inflammatory infiltrate consistent with PRP (Fig 4).

Her screening labs showed a positive QuantiFERON gold and positive Hepatitis C antibody. Due to rapid progression of the rash and severe ectropion from inflammation and edema of the face, she was admitted to the hospital for additional workup and wet wraps. A chest x-ray performed to rule out active TB found a nodule in the right upper lobe of her right lung concerning for a malignancy or infection. She was unable to produce sputum for an acid-fast bacilli stain. Ultimately, a biopsy of the nodule revealed T3N2M0 Stage 3 adenosquamous carcinoma of the lung.

In constellation with her skin findings, she was determined to have pPRP in the setting of newly diagnosed pulmonary adenosquamous carcinoma. Treatment options were limited due to latent TB, untreated chronic hepatitis C, and comorbid severe hypertension. The patient was started on clobetasol ointment used twice daily, home wet wraps of topical triamcinolone ointment, hydrocortisone ointment, and bland emollients in addition to acitretin 25 mg daily. Due to cost and perceived lack of benefit, the patient self-discontinued acitretin after 1 week. She did not receive oral prednisone. Despite persistent use of topical steroids, her rash continued to progress for several weeks. She ultimately underwent resection of her adenosquamous carcinoma of the right lung and treatment with wet wraps while hospitalized which resulted in remarkable rapid resolution or her rash.

## Discussion

Cutaneous paraneoplastic conditions are a diverse group of disorders that are related to an underlying internal malignancy.^5^ Cutaneous paraneoplastic manifestations often precede the diagnosis of malignancy, and dermatologists are therefore uniquely positioned to recognize these conditions, prompting a cancer work-up.^5^ pPRP is a relatively recently described cutaneous paraneoplastic condition that has been seen in association with several internal malignancies.

Including our case, there are 17 total cases of pPRP in the literature since 1983^6^ (Table I). Ages range from 26-89 years old, with 10 out of the 17 patients being male. Both solid organ and hematological malignancies are represented, with 14 (82%) being solid organ. Of 11 cases that showed complete or near resolution of pPRP, 6 (55%) had cancer-directed treatment only, 2 (18%) received cancer-directed treatment and skin-directed topical therapy, and 1 (9%) received treatment for cancer along with systemic retinoid treatment. Only 2 (18%) out of the 11 patients had resolution of pPRP without treating the underlying cancer. One patient received methotrexate monotherapy^15^ and another patient received topical steroids.^7^ The cases of pPRP in the literature since 1983 are summarized in the table below.

**Table I:** Summary of reported cases of paraneoplastic PRP in the literature

Patient	Cancer	Treatment				Outcome of skin disease
		Cancer-directed therapy	Systemic retinoids	Topicals	Other	
58 M^1^	Prostate carcinoma	--	Acitretin	Emollients; Keratolytics	--	Significant improvement
74 M^6^	Leukemia	--	--	Topical steroids	Oral prednisone	Moderate improvement
42 M^7^	Unknown primary with liver metastasis	--	--	Topical steroids; emollients	--	Complete resolution
73 M^8^	Kaposi sarcoma, melanoma, BCC	Surgical removal	--	Etretinate	--	Complete resolution
61 F^9^	Bronchogenic carcinoma	Radiotherapy	--	--	--	Complete resolution
76 M^10^	Renal cell carcinoma	Radical left nephrectomy	--	--	--	Complete resolution
46 M^11^	Laryngeal carcinoma	Left vocal cordectomy	--	--	--	Complete resolution
66 M^12^	Pulmonary adenocarcinoma	Lobectomy	--	--	--	Complete resolution
89 F^13^	Colonic adenocarcinoma	Surgical removal	--	--	--	Complete resolution
83 M^14^	Metastatic squamous cell carcinoma	Radiotherapy; Paclitaxel	--	--	--	Complete resolution
59 F^15^	Cholangiocarcinoma	--	--	--	Methotrexate	Complete resolution
72 F^16^	Myelodysplastic syndrome	Decitabine	--	Betamethasone	Urea	Near complete resolution
26 M^17^	Hepatocellular carcinoma	Intrahepatic doxorubicin	--	Topical vitamin A; emollients	--	Significant improvement
79 F^18^	Merkel cell, SCC	Radiotherapy	--	Etretinate	--	No improvement
75 F^19^	Metastatic adenocarcinoma	--	Acitretin	--	Prednisone	Temporary improvement
49 M^20^	Chronic lymphocytic leukemia	--	Acitretin	--	--	Unknown

## Conclusion

pPRP is a rare form of PRP associated with solid or hematolymphoid malignancies, which is often recalcitrate to standard treatments. Vitamin A analogues, oral steroids, and topical steroids treat symptoms, but the resolution of the rash usually requires treating underlying the malignancy. Evidence-based data on treatment are limited and largely drawn from case reports.^5^ The relationship between PRP and cancer's underlying mechanisms and the best approaches to treating both conditions remain to be identified.

Healthcare providers should consider cancer screening among patients with rapid-onset or treatment-resistant PRP. There are no current guidelines for malignancy workup recommendations in patients with PRP. Clinicians should start with age-appropriate cancer screening, a thorough review of systems, and evaluation of risk factors like a family history of cancer or use of tobacco or alcohol.

## Conflicts of interest

None disclosed.
